# Time for a sugary drinks tax in the UK?

**DOI:** 10.1093/pubmed/fdu033

**Published:** 2014-05-29

**Authors:** Oliver Mytton

**Affiliations:** UKCRC Centre for Diet and Activity Research (CEDAR), MRC Epidemiology Unit University of Cambridge School of Clinical Medicine, Box 285 Institute of Metabolic Science, Cambridge Biomedical Campus, Cambridge, CB2 0QQ

Food taxes are much discussed. However, the question for public health remains: what role could these taxes have in improving our health?

A response to the paper by Cornelsen *et al*.^1^ sets out some of the issues. While the authors' focus is on whether these taxes make us thin, others have argued the focus should be broader, being health or even the environment and health.^2,3^ The paper includes a valuable discussion of cross-price effects, the term economists give to the effect of food price changes on the purchases of other related food items. Such substitution effects can have an important role in determining the overall health impact of taxes on unhealthy foods. For example, taxing fatty foods might encourage people to switch to salty foods and this could off-set the beneficial health effects from reduced fat consumption.^4^ Understanding cross-price effects is also important, because their estimates are used to model the potential effect of food taxes. Cornelsen *et al*.^1^ set out some of the challenges in estimating these values, and their use in modelling studies.

The authors bring us up to date with latest evidence on existing food taxes, although what is really happening is sometimes hard to tell. Was cross-border shopping in Denmark really a major issue, or was it over-emphasized for political gain?^5^ Recent analysis also suggests that its primary goal of cutting purchasing of taxed items was successful in the short run, with a 10–15% drop in consumption of butter and fats, although its impact on overall diet was not quantified.^5^ Unfortunately, a full understanding of the tax will be difficult as it was only in place for less than a year.

More evaluation is needed. Indeed it is probably only through evaluation of actual taxes that we may address many of the issues and uncertainties that Cornelsen *et al*.^1^ raise. We need to be careful how we undertake and interpret any evaluation. The authors suggest that the ‘well-worn staples of public health’ are failing us. Herein may lie a danger that the success or failure of any one policy hinges on whether obesity rates rise or fall. We have a more nuanced approach to our evaluation of tobacco and alcohol control polices. For example, recent evaluations of plain packaging have focused on perceptions and thoughts to quit, often considering sub-groups within the population, not population health.^6^ For obesity, given its complexity and the diversity of influences on body weight,^7^ evaluation of interventions (including of food taxes) needs careful thought and consideration of how much of the signal can be detected amongst the noise.^8^

Where does this leave us in the UK? No health body is presently advocating a tax on unhealthy foods. This is partly because of the present uncertainties and concerns set out by Cornelsen *et al.*^1^ However, a tax on sugary drinks is supported by the Faculty of Public Health, Sustain, the UK Health Forum and the Academy of Medical Royal Colleges.^9,10^ This tax is a very different proposition to taxing food.

The evidence linking regular sugary drinks consumption to obesity, diabetes, cardiovascular disease and tooth decay comes from strong observational studies and, additionally for weight gain, from some randomized trials.^10^ The evidence is also underpinned by an understanding of the physiological and metabolic effects of sugary drinks on humans. Sugary drinks are non-necessities and can readily be replaced by water. Sugary drinks are un-satiating, resulting in over-consumption of calories and weight gain, and so appear a good target for removing excess calories from our diets. Empirical evidence suggests substitution with healthier drinks (e.g. diet drinks, bottled water, tea and coffee) is likely.^10^ This substitution may dampen the effect of a tax on sugary drinks, but even after allowing for these effects is forecast to reduce obesity numbers by around 180 000 adults (or 1.3% of obese adults), largely among younger adults, in the UK.^10^ It would also be helpful to understand its potential impact on other conditions, dental caries and diabetes.

A sugary drinks tax is likely to have other benefits. It will raise significant revenue that could be used to improve health.^11,12^ Its introduction may serve to heighten awareness of the health consequence of regular sugary drinks consumption.^9^ It may be a stimulus for beverage manufacturers to move away from full-sugar drinks to alternatives. The principle downside of the tax is that it would be regressive. Not that this has stopped public health bodies advocating for taxes on alcohol and tobacco and at the levels proposed the burden of the tax would be slight (around 9 pence per person per week).^10^

Obesity remains a wicked problem, and there is no one thing that will make us all thin.^13^ But that should not stop us backing sensible measures that look likely to help make some of us thinner and healthier.

## Funding

Funding to pay the Open Access publication charges for this article was provided by The University of Cambridge.
